# RhoG facilitates a conformational transition in the guanine nucleotide exchange factor complex DOCK5/ELMO1 to an open state

**DOI:** 10.1016/j.jbc.2024.107459

**Published:** 2024-06-08

**Authors:** Mutsuko Kukimoto-Niino, Kazushige Katsura, Yoshiko Ishizuka-Katsura, Chiemi Mishima-Tsumagari, Mayumi Yonemochi, Mio Inoue, Reiko Nakagawa, Rahul Kaushik, Kam Y.J. Zhang, Mikako Shirouzu

**Affiliations:** 1 Laboratory for Protein Functional and Structural Biology, RIKEN Center for Biosystems Dynamics Research, Yokohama, Kanagawa, Japan; 2 Drug Discovery Structural Biology Platform Unit, RIKEN Center for Biosystems Dynamics Research, Yokohama, Kanagawa, Japan; 3 Laboratory for Cell-Free Protein Synthesis, RIKEN Center for Biosystems Dynamics Research, Kobe, Hyogo, Japan; 4 Laboratory for Structural Bioinformatics, RIKEN Center for Biosystems Dynamics Research, Yokohama, Kanagawa, Japan

**Keywords:** electron microscopy (EM), guanine nucleotide exchange factor (GEF), protein structure, Rho (Rho GTPase), Ras-related C3 botulinum toxin substrate 1 (Rac1), signal transduction, single particle analysis, surface plasmon resonance (SPR)

## Abstract

The dedicator of cytokinesis (DOCK)/engulfment and cell motility (ELMO) complex serves as a guanine nucleotide exchange factor (GEF) for the GTPase Rac. RhoG, another GTPase, activates the ELMO-DOCK-Rac pathway during engulfment and migration. Recent cryo-EM structures of the DOCK2/ELMO1 and DOCK2/ELMO1/Rac1 complexes have identified closed and open conformations that are key to understanding the autoinhibition mechanism. Nevertheless, the structural details of RhoG-mediated activation of the DOCK/ELMO complex remain elusive. Herein, we present cryo-EM structures of DOCK5/ELMO1 alone and in complex with RhoG and Rac1. The DOCK5/ELMO1 structure exhibits a closed conformation similar to that of DOCK2/ELMO1, suggesting a shared regulatory mechanism of the autoinhibitory state across DOCK-A/B subfamilies (DOCK1−5). Conversely, the RhoG/DOCK5/ELMO1/Rac1 complex adopts an open conformation that differs from that of the DOCK2/ELMO1/Rac1 complex, with RhoG binding to both ELMO1 and DOCK5. The alignment of the DOCK5 phosphatidylinositol (3,4,5)-trisphosphate binding site with the RhoG C-terminal lipidation site suggests simultaneous binding of RhoG and DOCK5/ELMO1 to the plasma membrane. Structural comparison of the apo and RhoG-bound states revealed that RhoG facilitates a closed-to-open state conformational change of DOCK5/ELMO1. Biochemical and surface plasmon resonance (SPR) assays confirm that RhoG enhances the Rac GEF activity of DOCK5/ELMO1 and increases its binding affinity for Rac1. Further analysis of structural variability underscored the conformational flexibility of the DOCK5/ELMO1/Rac1 complex core, potentially facilitating the proximity of the DOCK5 GEF domain to the plasma membrane. These findings elucidate the structural mechanism underlying the RhoG-induced allosteric activation and membrane binding of the DOCK/ELMO complex.

Rho GTPases are key regulators of cytoskeletal dynamics that affect many cellular processes, such as morphogenesis, polarity, motility, and cell division (1). Dedicator of cytokinesis (DOCK) proteins are evolutionarily conserved guanine nucleotide exchange factors (GEFs) for Rho GTPases Rac and Cdc42, facilitating the conversion of these GTPases from GDP- to GTP-bound forms (2, 3). Eleven mammalian DOCK proteins regulate various normal and pathological processes of development and immunity by activating Rac and Cdc42 (4, 5).

Proteins in the DOCK family are classified into four subfamilies (A−D) based on sequence homology, each possessing two shared domains: dock homology region (DHR)-1 and DHR-2. DHR-1 binds to phosphoinositide (6), whereas DHR-2 mediates GEF activity and homodimerization (2, 7). The DOCK-A (DOCK1/2/5) and DOCK-B (DOCK3/4) subfamilies specifically activate Rac and have an Src homology domain at their N-terminus; the Src homology domain is involved in autoinhibition (8) and binding to engulfment and cell motility (ELMO) scaffold proteins (9, 10). ELMO is structurally divided into two domains: an N-terminal domain (NTD), consisting of the Ras-binding (RBD), ELMO inhibitory (EID) (11), and ELMO domains, and a C-terminal domain (CTD), consisting of the Pleckstrin homology (PH) domain and proline-rich region (Fig. 1*A*). The CTD of ELMO1 enhances the GEF activity of DOCK1 for Rac1 (12), whereas the NTD is essential for DOCK1-mediated intracellular Rac activation (13). ELMO^NTD^ mediates interactions with upstream proteins such as the Rho GTPase RhoG (14), the Arf GTPase Arl4A (15), and the BAI G-protein-coupled receptors (16, 17). RhoG activates Rac1 through ELMO/DOCK to control cell morphology, engulfment, and cell migration (14, 18, 19, 20).

**Figure 1:**
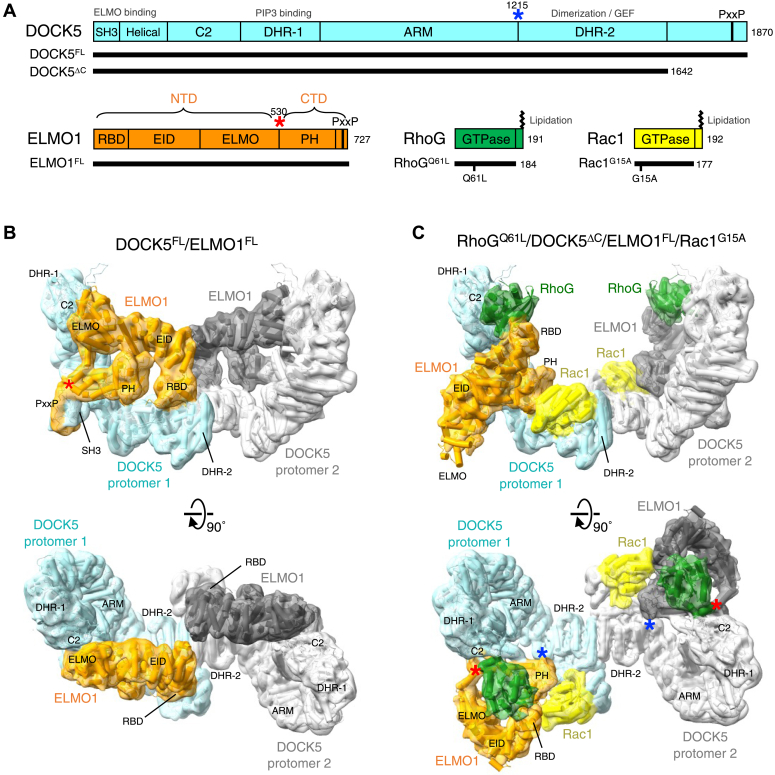
**Structures of the DOCK5/ELMO1 complexes**. *A*, a schematic diagram of the domain boundaries of human DOCK5, ELMO1, RhoG, and Rac1. *Black bars* indicate the protein constructs used in this study. *Asterisks* (DOCK5, *blue*; ELMO1, *red*) indicate the hinges of the conformational changes observed in this study. *B* and *C*, Cryo-EM density (composite) maps and atomic models of the DOCK5/ELMO1 (*B*) and RhoG/DOCK5/ELMO1/Rac1 (*C*) complexes in two orthogonal views. *Asterisk*s indicate the location of the hinges shown in *A*. ARM, armadillo repeat domain; DHR: dock homology region; EID, Elmo inhibitory domain; ELMO, Elmo domain; PH, Pleckstrin homology domain; PxxP, proline-rich region; RBD, Ras-binding domain; SH3, Src homology domain 3.

Recent cryo-EM structures of the DOCK2/ELMO1 complex in the Rac1-bound and unbound states revealed that autoinhibitory regulation occurs through conformational changes in the ELMO1^NTD^ (21). In addition, the crystal structures of the RhoG/ELMO^RBD^ and BAI1/ELMO2^NTD^ complexes were determined (21, 22, 23). Based on these structures, it was hypothesized that the binding of RhoG and/or BAI1 to ELMO1^NTD^ would release DOCK2/ELMO1 from autoinhibition and promote its localization to the plasma membrane. However, the structure of the DOCK/ELMO complex bound to RhoG and other upstream proteins has not yet been elucidated.

Our previous study illustrated the cryo-EM structure of the DOCK5/ELMO1/Rac1 complex (24), which is a more curved dimer than the DOCK2/ELMO1/Rac1 complex. However, the significance of the observed structural difference remains unclear. ELMO1^NTD^ appeared disordered in the DOCK5/ELMO1/Rac1 complex, whereas in the DOCK2/ELMO1/Rac1 complex, it was structured into an S-shaped open conformation (21). In the S-shaped conformation of ELMO1^NTD^, the symmetric DOCK2/ELMO1 dimer and RhoG cannot simultaneously interact with the membrane (25). Therefore, it remains unclear how the DOCK/ELMO complex is activated by membrane-localized RhoG. To address these questions, we determined the cryo-EM structures of the DOCK5/ELMO1 complex alone and bound to RhoG and Rac1.

## Results and discussion

### The structure of DOCK5/ELMO1

We initially determined the cryo-EM structure of the DOCK5/ELMO1 binary complex using human full-length DOCK5 (DOCK5^FL^, residues 1−1870) and ELMO1 (ELMO1^FL^, residues 1−727) proteins (Fig. 1*A*). Our first attempt to analyze the DOCK5^FL^/ELMO1^FL^ complex produced a low-resolution (7.3 Å) cryo-EM map with two-fold symmetry but lacked density for ELMO1^NTD^ (Fig. S1). We next analyzed a DOCK5^FL^/ELMO1^FL^ sample crosslinked with bis(sulfosuccinimidyl)suberate (BS^3^), a widely used N-hydroxysuccinimide ester-based crosslinking agent (26), achieving a reconstruction at improved resolution (5.8 Å) that clearly showed the ELMO1^NTD^ density with two-fold symmetry (Figs. S1 and S2). To enhance local resolution, we symmetrically expanded and refined the particles, focusing on a single DOCK5/ELMO1 protomer and the dimerization component, which led to structure determination at 4.8 Å resolution (Fig. S2 and Table S1). Model building and refinement of the DOCK5^FL^/ELMO1^FL^ complex were completed, with the exception of the C-terminal region of DOCK5 (residues 1643−1870), which remained disordered.

The structure of DOCK5/ELMO1 showed a closed conformation similar to that reported for DOCK2/ELMO1 (21) (Fig. 1*B*). The α-solenoid of ELMO1^NTD^ was clamped onto the DHR-2 domain of DOCK5, occluding the Rac1 binding site. This indicates that the DOCK1−5/ELMO1 complexes likely have autoinhibitory regulation in common.

**Figure 2:**
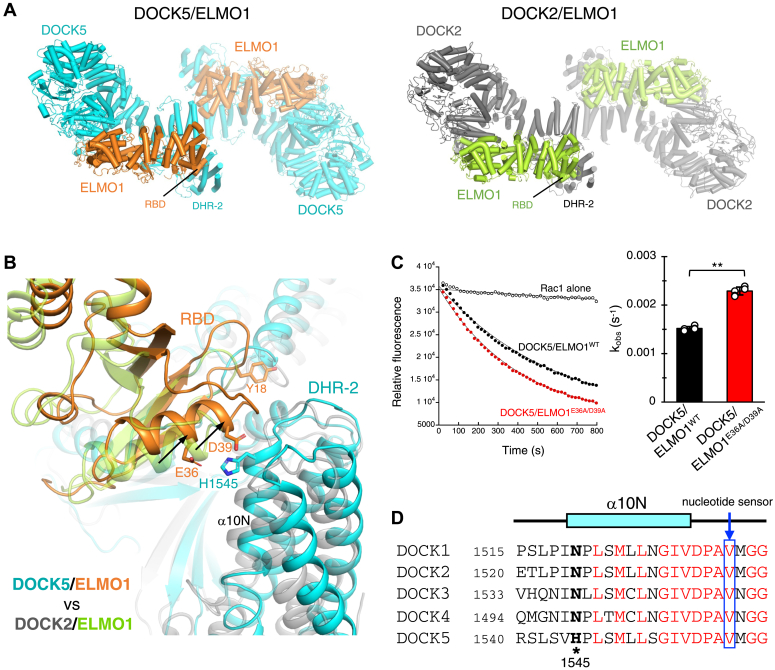
**Autoinhibitory interactions in DOCK5/ELMO1**. *A*, comparison of the structures of the DOCK5/ELMO1 (*left*) and DOCK2/ELMO1 (PDB ID: 6TGB, *right*) complexes, looking down the 2-fold symmetry axis. In each structure, the protomers on the right are shown as transparent. *B*, interactions between ELMO1 RBD and DHR-2 in DOCK5/ELMO1 (*orange* and *cyan*) superimposed with the corresponding structure of DOCK2/ELMO1 (*gray and lemon, transparent*). *C*, GEF activity of DOCK5/ELMO1^WT^ and DOCK5/ELMO1^E36A/D39A^ against Rac1. Data are presented as mean ± SD (n = 2 independent experiments with 3 technical replicates; unpaired two-sided Student’s *t* test, ∗∗*p* = 0.00744). *D*, sequence alignment of DOCK1−5 around the α10N helix and the catalytic nucleotide sensor.

Despite the common closed conformation, a detailed structural comparison revealed that DOCK5/ELMO1 is more compact than DOCK2/ELMO1, with the two ELMO1 NTDs located closer to each other (Fig. 2*A*). A closer interaction was observed for DOCK5 than for DOCK2 at the ELMO1^RBD^-DHR-2 interface, between Glu36 and Asp39 in ELMO1 and His1545 in DOCK5 (Fig. 2*B*). The observed differences are unlikely to be due to the crosslinking of DOCK5/ELMO1, as crosslinked samples were similarly used in the cryo-EM analysis of DOCK2/ELMO1 (21). We performed structure-based mutagenesis at the ELMO1^RBD^-DOCK5^DHR-2^ interface to examine the GEF activity of DOCK5^FL^/ELMO1^FL^ against Rac1 (GTPase domain, residues 1−177). Simultaneous mutation of Glu36 and Asp39 in ELMO1 to alanine (DOCK5/ELMO1^E36A/D39A^) resulted in a 1.5-fold increase in GEF activity for Rac1 compared to DOCK5/ELMO1^WT^ (Fig. 2*C*). This suggests that these ELMO1 residues are involved in the DOCK5/ELMO1 autoinhibitory interaction. His1545 in DOCK5 is not conserved in the DOCK-A/B subfamilies from DOCK1 to DOCK4 (Fig. 2*D*), suggesting that the autoinhibitory conformation may be specifically stabilized in DOCK5.

### RhoG activates DOCK5/ELMO1 by promoting its binding to Rac1

**Figure 3:**
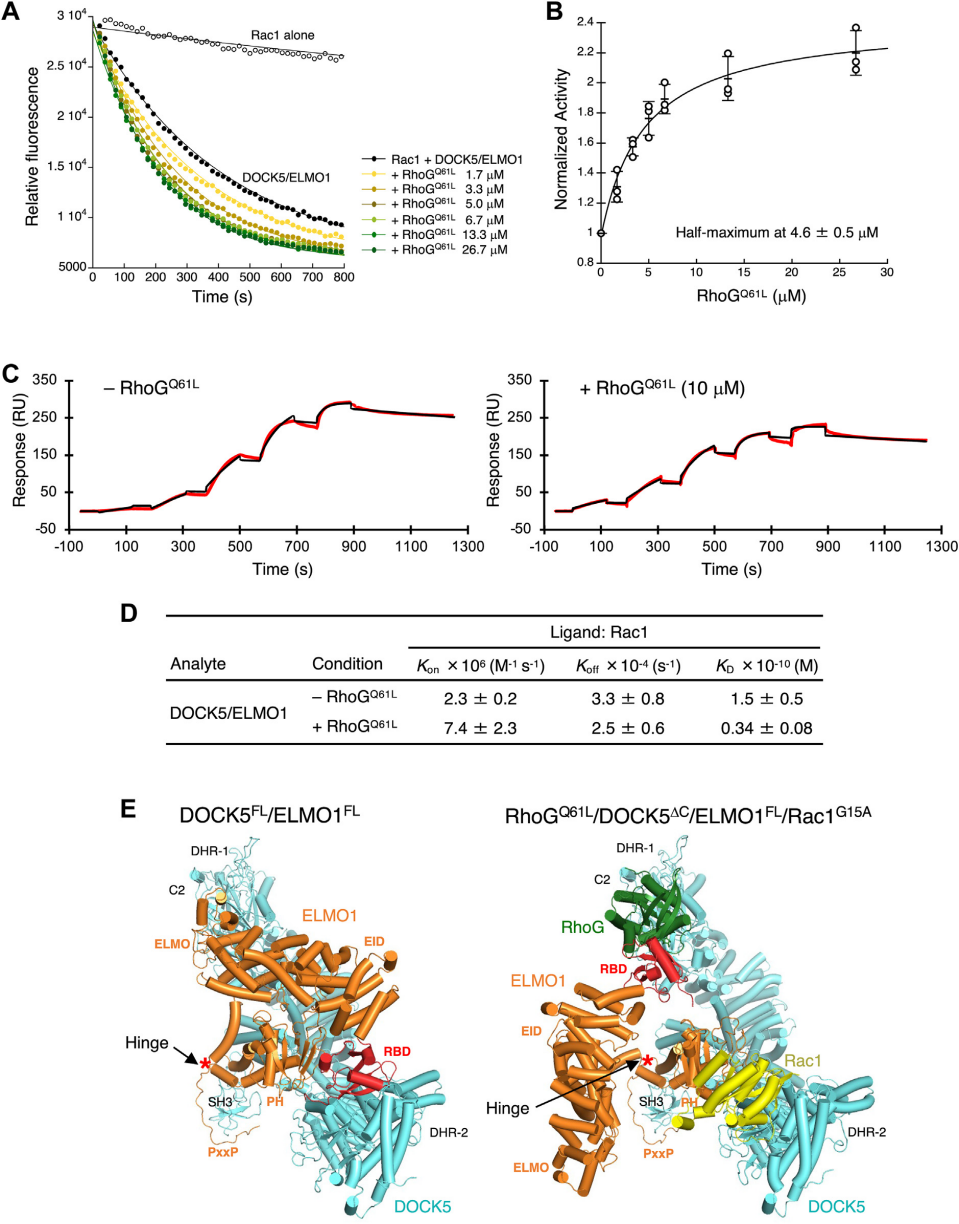
**Biochemical characterization and conformational changes of DOCK5/ELMO1**. *A*, GEF activity of DOCK5/ELMO1 against Rac1 with the addition of indicated concentrations of RhoG^Q61L^. *B*, effects of RhoG^Q61L^ concentration on the biochemical activity of DOCK5/ELMO1 analyzed from the experiments shown in *A*. The activity was normalized relative to that at 0 μM RhoG^Q61L^. Data are presented as mean ± SD (n = 3 independent experiments). *C*, binding of DOCK5/ELMO1 to Rac1-immobilized SPR biosensor in the absence (*left*) and presence of RhoG^Q61L^ at 10 μM (*right*). *D*, kinetic parameters of DOCK5/ELMO1 binding to Rac1 estimated from the experiments shown in *C*. *k*_on_, association rate constant; *k*_off_, dissociation rate constant; *K*_D_ = k_off_/k_on_, equilibrium dissociation constant. Data are presented as mean ± SD (n = 3 independent experiments). *E*, a comparison of the protomer structures of DOCK5/ELMO1 (*left*) and RhoG/DOCK5/ELMO1/Rac1 (*right*). DOCK5 is depicted in cyan, ELMO1 in orange except for the *red* RBD, Rac1 in *yellow*, and RhoG in *green*.

Next, we examined the GEF activity of DOCK5^FL^/ELMO1^FL^ against Rac1 in the presence of the constitutively active RhoG^Q61L^ mutant (GTPase domain, residues 1−184). The GEF activity of DOCK5^FL^/ELMO1^FL^ increased with increasing RhoG^Q61L^ concentration, reaching half-maximum at 4.6 μM (Fig. 3, *A* and *B*). This value is in close agreement with the binding affinity (K_D_ = 10 μM) of RhoG^Q61L^ for DOCK5^ΔC^/ELMO1^FL^, a complex of C-terminal truncated DOCK5 (DOCK5^ΔC^, residues 1−1642) and full-length ELMO1 (Fig. 1*A*), as determined by the surface plasmon resonance (SPR) (24), indicating that DOCK5/ELMO1 is activated by binding to the active form of RhoG, as expected.

We also used SPR to examine the effect of RhoG^Q61L^ on the Rac1 binding affinity for DOCK5^FL^/ELMO1^FL^. We immobilized Rac1 (residues 1−188) on an SPR sensor chip and passed varying concentrations of DOCK5^FL^/ELMO1^FL^ in the absence or presence of 10 μM RhoG^Q61L^ to measure the rates of association and dissociation (Fig. 3*C*). DOCK5^FL^/ELMO1^FL^ showed a higher affinity for Rac1 in the presence of RhoG^Q61L^ (*K*_D_ = 0.034 nM) than in its absence (*K*_D_ = 0.15 nM) (Fig. 3*D*). Collectively, these results suggest that RhoG activates DOCK5/ELMO1 by facilitating its binding to Rac1.

### The structure of the RhoG/DOCK5/ELMO1/Rac1 complex

We conducted cryo-EM single particle analysis on the RhoG/DOCK5/ELMO1/Rac1 complex to elucidate the structural basis of DOCK5/ELMO1 activation by RhoG. The components used in this analysis included DOCK5^ΔC^, ELMO1^FL^, RhoG^Q61L^, and the nucleotide-deficient Rac1^G15A^ mutant (GTPase domain, residues 1−177) (Fig. 1*A*). Size exclusion chromatography yielded a stable DOCK5^ΔC^/ELMO1^FL^/Rac1^G15A^ complex (24). Conversely, SPR analysis showed that RhoG^Q61L^ and DOCK5^ΔC^/ELMO1^FL^ readily dissociated (24). Therefore, to define the RhoG-bound structure, we analyzed the crosslinked DOCK5^ΔC^/ELMO1^FL^/Rac1^G15A^ complex in the presence of excess RhoG^Q61L^ using BS^3^.

The cross-linked RhoG^Q61L^/DOCK5^ΔC^/ELMO1^FL^/Rac1^G15A^ sample yielded a two-fold symmetrical reconstruction at a resolution of 3.8 Å (Fig. S3). However, the ELMO1^NTD^ was poorly resolved due to its high mobility. To address this, we symmetrically expanded and refined the particles focusing on ELMO1 NTD (Fig. S3). Through 3D classification, excluding the DOCK5 DHR-2/Rac1 segment, ELMO1^NTD^ was resolved into several conformations. One class, comprising approximately 12% of the particles, provided a map of sufficient quality to define the structure of DOCK5/ELMO1 bound to RhoG at 4.9 Å resolution (Fig. S3 and Table S1). Cross-linking mass spectrometry confirmed RhoG’s placement within the cryo-EM map (Fig. S4 and Table S2). From this partial map, we reconstructed the entire RhoG/DOCK5/ELMO1/Rac1 complex at 4.7 Å resolution with an ordered RhoG-ELMO1^NTD^ (Fig. S5 and Table S1). Further reconstruction with both ordered RhoG-ELMO1^NTD^ yielded similar maps at resolutions of 8.2 Å and 7.3 Å with C1 and C2 symmetry, respectively (Fig. S5).

**Figure 4:**
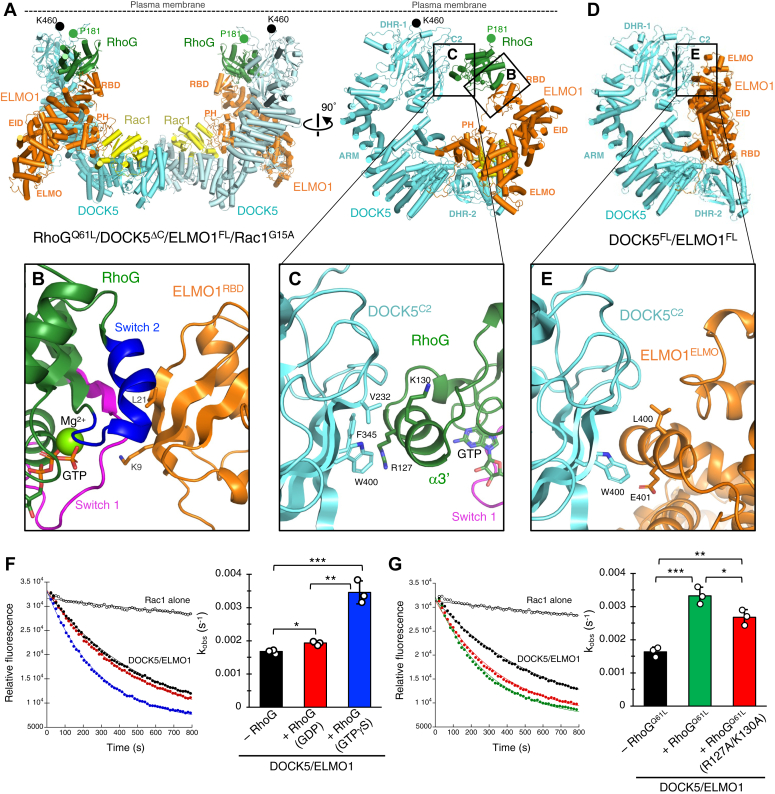
**RhoG interaction sites in DOCK5/ELMO1**. *A*, structure of the RhoG/DOCK5/ELMO1/Rac1 complex from two orthogonal views. The locations of membrane-binding sites (the DOCK5 PIP3-binding residue Lys460 and the RhoG C-terminus Pro181) are indicated by *black* and *green circles*, respectively. *B*, a close-up view of the RhoG-ELMO1^RBD^ interface boxed in *A*. RhoG is shown in *green* except for switch 1 (*magenta*) and switch 2 (*blue*). *C*, a close-up view of the DOCK5^C2^-RhoG interface boxed in *A*. *D*, structure of the DOCK5/ELMO1 complex. The molecular orientation of DOCK5 is the same as the right panel in *A*. *E*, a close-up view of the DOCK5^C2^-ELMO1^ELMO^ interface boxed in *D*. *F*, GEF activity of DOCK5/ELMO1 against Rac1 with the addition of 16 μM GDP-bound or GTPγS-bound RhoG^WT^. Data are presented as mean ± SD (n = 3 independent experiments; unpaired two-sided Student’s *t* test, ∗*p* = 0.00673, ∗∗*p* = 0.00168, ∗∗∗*p* = 0.000933). *G*, effects of the R127A/K130A mutation in RhoG^Q61L^ on DOCK5/ELMO1 GEF activity against Rac1. Each RhoG protein was added to the reaction at 6.7 μM. Data are presented as mean ± SD (n = 3 independent experiments; unpaired two-sided Student’s *t* test, ∗*p* = 0.0323, ∗∗*p* = 0.00264, ∗∗∗*p* = 0.00225).

In the RhoG-bound DOCK5^ΔC^/ELMO1^FL^/Rac1^G15A^ complex, a curved dimer formed, with RhoG located at both protomer tips (Fig. 1*C*). Rac1 interacted with the DHR-2 domain of DOCK5 and the PH domain of ELMO1 near the dimer interface, akin to the prior DOCK5^ΔC^/ELMO1^FL^/Rac1^G15A^ ternary complex (24). Notably, ELMO1^NTD^ was disordered in the previous ternary structure. However, in the present RhoG-bound structure, the α-solenoid of ELMO1^NTD^ arched toward the DHR-1 domain of DOCK5 and bound to RhoG in cooperation with the C2 domain of DOCK5 (Fig. 1*C*). Due to this binding mode, the ELMO1^NTD^ of the RhoG/DOCK5/ELMO1/Rac1 complex had a compact, open conformation that was different from the elongated (S-shaped) open conformation of the DOCK2/ELMO1/Rac1 complex (Fig. S6). In the present RhoG/DOCK5/ELMO1/Rac1 structure, the C-terminus of RhoG, Pro181, near the lipidation site, is adjacent to the phosphatidylinositol (3,4,5)-trisphosphate binding site of DOCK5, which includes basic residues such as Lys457, Lys460, and Lys464 (Fig. 4*A*). Four membrane-binding sites (two DHR-1 and two RhoG) were aligned in the same direction in the dimeric configuration. Thus, the present structure suggests that RhoG and the symmetric DOCK5/ELMO1 dimer can simultaneously bind to the plasma membrane.

Comparison of the apo (DOCK5/ELMO1) and RhoG-bound (RhoG/DOCK5/ELMO1/Rac1) complexes revealed that ELMO1^NTD^ rotates approximately 120° and swings out toward the DOCK5 C2/DHR-1 domains (Fig. 3*E* and Movie S1). RhoG interacted with ELMO1^RBD^ on one side and with DOCK5^C2^ on the other side to mediate an open conformation of DOCK5/ELMO1. These structures suggest that RhoG facilitates the conformational change of DOCK5/ELMO1 from the closed to the open state, thereby relieving the Rac1-binding site in DOCK5^DHR-2^ from autoinhibition, consistent with the results of biochemical and SPR binding analyses (Fig. 3, *A*−*D*).

### RhoG-binding interface of DOCK5/ELMO1

RhoG^Q61L^ formed a primary interface (∼440 Å^2^ buried surface area) with the ELMO1^RBD^ and a secondary interface (∼260 Å^2^ buried surface area) with DOCK5^C2^ (Fig. 4, *A*−*C*). Consistent with the previous crystal structures of the RhoG-ELMO^RBD^ complexes (21, 23), the primary interface involved ELMO1 residues such as Lys9 and Leu21 interacting with the switch 1 and 2 regions of RhoG^Q61L^ in the GTP-bound conformation (Fig. 4*B*). This is consistent with the fact that DOCK5^ΔC^/ELMO1^FL^ interacted with RhoG^Q61L^ but not with the GDP-bound RhoG (24). By contrast, the secondary interface consisted of aromatic or aliphatic amino acid residues, such as Val232, Phe345, and Trp400 of DOCK5, which interacted with Arg127 and Lys130 of the Rho insertion region (α3′ helix) of RhoG^Q61L^ (Fig. 4*C*). Thus, the secondary interface appears to be independent of the nucleotide-binding state of RhoG. The C2 domain of DOCK5 interacted with the ELMO domain of ELMO1 in the apo state of DOCK5/ELMO1 (Fig. 4, *D* and *E*). Therefore, DOCK5^C2^ may have broad specificity in protein–protein interactions.

We performed biochemical experiments to evaluate the RhoG binding interface observed in the cryo-EM structure. Because the RhoG-ELMO1^RBD^ interaction is constituted by the RhoG switch regions that undergo nucleotide-dependent conformational changes (Fig. 4*B*), we first assessed the ability of GDP-bound or GTPγS-bound RhoG^WT^ to activate DOCK5^FL^/ELMO1^FL^ in an *in vitro* GEF assay (Fig. 4*F*). GTPγS-bound RhoG enhanced the GEF activity against Rac1 in DOCK5^FL^/ELMO1^FL^ by 2.06-fold, whereas GDP-bound RhoG enhanced it only slightly (by 1.15-fold) as expected. This result confirmed the importance of the RhoG-ELMO1^RBD^ interaction in the activation of DOCK5/ELMO1 by RhoG.

Next, we assessed the RhoG-DOCK5^C2^ interaction by structure-based mutagenesis: Arg127 and Lys130 of RhoG^G61L^ at the DOCK5 interface were simultaneously mutated to alanine (R127A/K130A) (Fig. 4*C*), and its ability to activate DOCK5^FL^/ELMO1^FL^ was examined. The control RhoG^Q61L^ enhanced the GEF activity of DOCK5^FL^/ELMO1^FL^ by 2.07-fold, whereas the R127A/K130A mutant of RhoG^Q61L^ enhanced it by 1.67-fold, a 20% reduction compared to RhoG^Q61L^ (Fig. 4*G*). These results indicate that the RhoG-DOCK5^C2^ interaction contributes to the activation of DOCK5/ELMO1 by RhoG in an *in vitro* system.

### DOCK5/ELMO1/Rac1 flatter conformation

**Figure 5:**
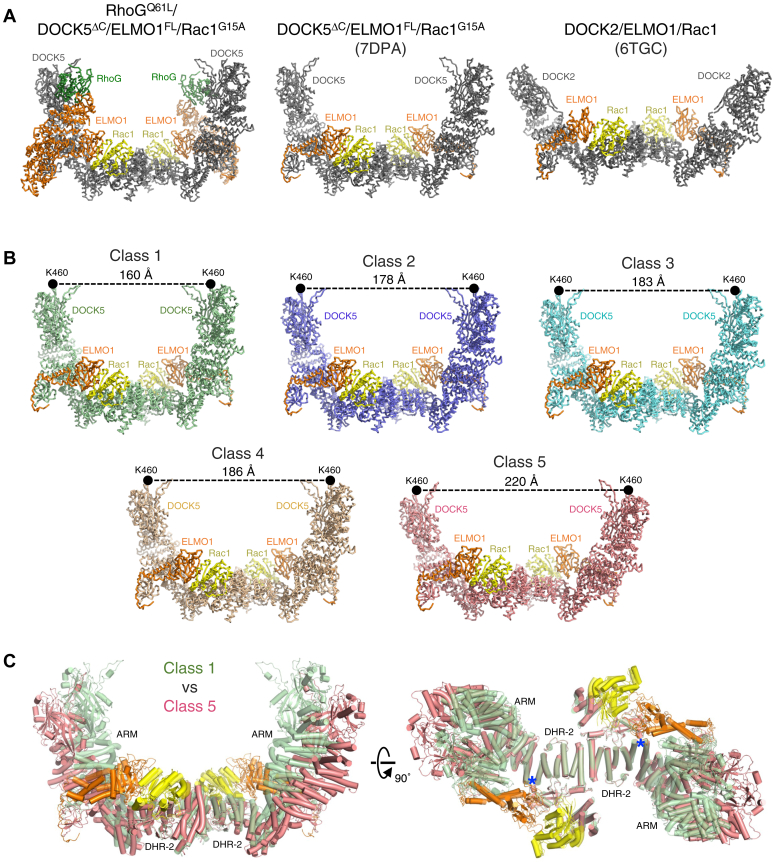
**Structural plasticity in DOCK5**. *A*, comparison of the RhoG/DOCK5/ELMO1/Rac1 complex (this study, *left*) with the DOCK5/ELMO1/Rac1 complex (PDB ID: 7DPA) and the DOCK2/ELMO1/Rac1 complex (PDB ID: 6TGC, ELMO1^NTD^ omitted for clarity). *B*, comparison of five classes of the DOCK5/ELMO1^CTD^/Rac1 core structures. The location of the Lys460 residue of DOCK5 in each protomer is indicated by a *black circle*. *C*, structural superimposition of two of five classes (class 1 and class 5 with the same color scheme as in *B*, class 1 is shown as *transparent*) in two orthogonal views. *Blue asterisks* indicate the location of the hinge between ARM and DHR-2 in DOCK5.

The RhoG-bound DOCK5/ELMO1/Rac1 dimer exhibited a similar curved structure as the unbound DOCK5/ELMO1/Rac1, while the DOCK2/ELMO1/Rac1 dimer exhibited a flatter structure (Fig. 5*A*). We thus evaluated conformational heterogeneity as a possible cause of these structural differences. We performed 3D variability analysis using the 3.8 Å-resolution consensus map of RhoG/DOCK5/ELMO1/Rac1. This analysis revealed several continuous movements within individual protomers of the DOCK5/ELMO1^CTD^/Rac1 core region (Movies S2 and S3). The major movement was that DOCK5 showed flexibility between N- and C-terminal modules at the boundary between armadillo repeat domain and DHR-2.

Next, to capture representative conformations of the DOCK5/ELMO1^CTD^/Rac1 core region, we divided the RhoG/DOCK5/ELMO1/Rac1 consensus map into 10 classes by 3D classification (Fig. S7). Five classes of sufficient quality were obtained with resolutions ranging from 4.2 to 4.5 Å and subjected to atomic model building and refinement (Table S3). The resulting structures of classes 1 to 5 differed from one another in dimeric molecular dimensions (Fig. 5*B*). The distance between the two DHR-1 of DOCK5 at the tip of the DOCK5/ELMO1^CTD^/Rac1 protomers, measured between the Cα atoms of Lys460 (one of the phosphatidylinositol (3,4,5)-trisphosphate binding residues), was shortest for class 1 and longest for class 5. Structural differences were due to the conformational changes between the armadillo repeat domain and DHR-2 in DOCK5, accompanied by a shift in the PH domain of ELMO1 (Fig. 5*C*). Of the five, class 5 of DOCK5/ELMO1^CTD^/Rac1 showed a flatter dimer than that of the others, similar to the DOCK2/ELMO1/Rac1 dimer (Fig. 5, *A* and *B*). Collectively, these results suggest that DOCK2/ELMO1 and DOCK5/ELMO1 are intrinsically similar in structure and exhibit conformational flexibility.

Our previous study of the DOCK5/ELMO1/Rac1 ternary complex (without RhoG) focused on obtaining the highest resolution map for *de novo* model building and did not detect conformational flexibility. However, since RhoG binding has little effect on the overall conformation of the DOCK5/ELMO1/Rac1 core region (Fig. 5*A*), it is possible that conformational changes similar to those in this study could occur even without RhoG binding.

**Figure 6:**
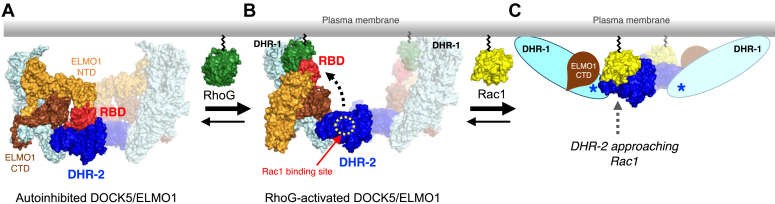
**A proposed structural model of RhoG-mediated DOCK5/ELMO1 activation**. *A*, the DOCK5/ELMO1 dimer localizes to the cytoplasm in the resting state. A subpopulation of DOCK5/ELMO1 is in a closed conformation in which the Rac1-binding site is occluded by the ELMO1 RBD. *B*, when RhoG is activated, the GTP-bound form of RhoG binds to ELMO1 RBD, shifting DOCK5/ELMO1 into an open conformation with the Rac1 binding site exposed. *C*, a hypothetical model of the DOCK5/ELMO1^CTD^ core approaching Rac1. A conformational change at the DOCK5 hinge (between ARM and DHR-2, *blue asterisks*) would bring DHR-2 closest to membrane-localized Rac1 for the GEF reaction. In each panel, the protomers on the *right* are shown as transparent.

In summary, the structural, biochemical, and biophysical analyses presented here reveal the RhoG-regulated allosteric mechanism of DOCK5/ELMO1 activation. Although this study is based on a simplified system using soluble RhoG and Rac1 proteins lacking C-terminal lipidation, the binding mode of RhoG suggests that DOCK5/ELMO1 activation occurs at the plasma membrane (Fig. 6). In contrast, the Rac1 binding site in DOCK5/ELMO1 is located in the concavity of the curved dimer, away from the predicted membrane surface. This suggests that the DHR-2 domain must be substantially rearranged during the membrane recruitment of DOCK5/ELMO1 to enable binding to membrane-localized Rac1. Consistent with this theory, our cryo-EM single particle analysis reveals that the DOCK5/ELMO1^CTD^/Rac1 core region is highly flexible and can, therefore, readily transition to a flatter conformation. We hypothesize that the flexibility observed in DOCK5 may allow the DHR-2 domain to approach the membrane-localized Rac under intracellular conditions. The lack of comprehensive knowledge of the conformational changes in the DOCK/ELMO complex on the membrane is attributed to limitations associated with current soluble protein-based experimental strategies. Structural analysis using lipid membranes and lipidated Rho GTPases will pave the way for understanding the molecular mechanisms underlying DOCK-mediated Rac activation in cells.

## Experimental procedures

### Preparation of the DOCK5/ELMO1 complex

The DOCK5/ELMO1 complexes were prepared as previously described (24). Briefly, DOCK5^FL^ (residues 1−1870) or DOCK5^ΔC^ (residues 1−1642) with N-terminal FLAG and streptavidin-binding peptide (SBP) tags were co-expressed with ELMO1^FL^ (residues 1−727) with an N-terminal FLAG tag in FreeStyle 293-F cells. Each resulting DOCK5/ELMO1 complex was purified using Streptavidin Sepharose beads (Cytiva), tag digestion with TEV protease, and size-exclusion chromatography on a HiLoad 16/600 Superose 6 pg column (Cytiva). For cryo-EM of the apo-complex, DOCK5^FL^/ELMO1^FL^ was crosslinked with 1 mM BS^3^ for 15 min at 25 °C, quenched with 50 mM Tris-HCl at pH 8.0, and purified through size-exclusion chromatography on a HiLoad 16/600 Superose 6 pg column in 20 mM HEPES-NaOH (pH 8.0), 300 mM NaCl, and 1 mM TCEP.

### Preparation of the RhoG^Q61L^/DOCK5^ΔC^/ELMO1^FL^/Rac1^G15A^ complex

Rac1-G15A (residues 1−177) and RhoG-Q61L mutants (residues 1−184) with an N-terminal His tags were expressed using the *Escherichia coli* cell-free protein synthesis system (27, 28) and purified through Ni-NTA affinity chromatography, tag digestion with TEV protease, and finally through size-exclusion chromatography as previously described (24). For the purification of RhoG^Q61L^ and its mutant (R127A/K130A) proteins, 1 mM MgCl_2_ and 10 μM GTP were added during the process. The purified DOCK5^ΔC^/ELMO1^FL^ complex was mixed with Rac1^G15A^, incubated overnight at 4 °C, and then loaded onto size-exclusion chromatography on a HiLoad 16/600 Superose 6 pg column in 20 mM Hepes-NaOH buffer (pH 8.0), 300 mM NaCl, and 1 mM TCEP. The resulting DOCK5^ΔC^/ELMO1^FL^/Rac1^G15A^ complex (0.9 μM) was mixed with RhoG^Q61L^ (15 μM), incubated overnight at 4 °C, and cross-linked with 1 mM BS^3^ for 15 min at 25 °C. After quenching the mixture with 50 mM Tris-HCl at pH 8.0, excess RhoG^Q61L^ was separated from the crosslinked complex using size-exclusion chromatography on a HiLoad 16/600 Superose 6 pg column in 20 mM HEPES-NaOH (pH 8.0), 300 mM NaCl, and 1 mM TCEP. Peak fractions were concentrated and used for cryo-EM analysis. The same sample was also analyzed through crosslinking mass spectrometry using a previously described method (24).

### Cryo-EM sample preparation, data collection, and data processing

Three microliters of protein sample (diluted to 200−400 nM in 20 mM HEPES-NaOH pH 8.0, 300 mM NaCl, 1 mM TCEP, and 0.06% digitonin) were applied to the glow-discharged holey carbon grids (Quantifoil R1.2/1.3, Cu, 300 mesh) supported with a thin film of carbon or graphene. Grids were incubated for 30 s at 4 °C and 100% humidity, blotted for 1 s, and plunge-frozen in liquid ethane using a Vitrobot Mark IV (Thermo Fisher Scientific). The data were collected on a 300 kV Titan Krios G4 electron microscope (Thermo Fisher Scientific) using a K3 direct electron detector (Gatan) in counting mode. Micrograph movies were acquired at a nominal magnification of 64,000×, corresponding to a calibrated pixel size of 1.33 Å per pixel. Each movie was recorded for 4.1 s and subdivided into 50 or 48 frames. The electron flux rate was set to 21.4 to 21.8 e^−^ per pixel/second at the detector, resulting in an accumulated exposure of 50 e^−^/Å^2^ at the specimen. The data were automatically acquired through the image shift method using EPU software with a defocus range of −0.8 to −2.0 μm. The collected movies were motion-corrected using MotionCor2 (29) with dose weighting. Parameters of the contrast transfer function were estimated from the motion-corrected micrographs using CTFFIND4 (30). Particles were automatically picked using crYOLO (31), and subsequent 2D and 3D analyses were performed using Relion 3.1 (32). Detailed data processing schemes for the DOCK5^FL^/ELMO1^FL^ and RhoG^Q61L^/DOCK5^ΔC^/ELMO1^FL^/Rac1^G15A^ complexes are shown in Figs S2–S5, respectively. Moreover, 3D continuous conformational heterogeneity was evaluated by 3D variability analysis in cryoSPARC (33).

### Model building and refinement

The atomic model was built using the structures of the DOCK5^ΔC^/ELMO1^FL^/Rac1^G15A^ complex (PDB ID: 7DPA) and the ELMO1^RBD^-RhoG complex (PDB ID: 7Y4A). The AlphaFold2 model (34) was utilized to further improve DOCK5 sequence assignments, especially in the C2 domain. The ELMO1 region 82−530 was modeled based on sequence homology with the corresponding region of ELMO2 (Protein Data Bank (PDB) ID: 6IE1). The model was fitted to the cryo-EM density map as a rigid body using UCSF Chimera (35) and manually adjusted using COOT (36). The final model was refined using Phenix (37).

### *In vitro* GEF assays

All measurements of the nucleotide exchange reaction were performed using the full-length DOCK5^FL^/ELMO1^FL^ complex and the Rac1 GTPase domain (residues 1−177). For DOCK5/ELMO1^WT^ or DOCK5/ELMO1^E36A/D39A^ GEF assays, each DOCK5/ELMO1 (25 nM) or control buffer was incubated with fluorescent boron-dipyrromethene-fluor (BODIPY-FL)-GDP-loaded Rac1^WT^ (1.6 μM) in reaction buffer containing 100 μM GTP, 20 mM Tris-HCl (pH 8.0), 150 mM NaCl, 10 mM MgCl_2_, and 0.2 mg/ml BSA. For the DOCK5^FL^/ELMO1^FL^ GEF assay, upon addition of RhoG proteins, 25 nM DOCK5^FL^/ELMO1^FL^ was premixed with indicated concentrations of RhoG^Q61L^ (1.7, 3.3, 5.0, 6.7, 13.3, and 26.7 μM) or 16 μM RhoG^WT^ (GDP- and GTPγS-bound forms) and incubated with 1.6 μM BODIPY-FL-GDP-loaded Rac1^WT^ in reaction buffer containing 100 μM GTP, 20 mM Tris-HCl (pH 8.0), 150 mM NaCl, and 5 mM MgCl_2_. The release of BODIPY-FL-GDP by Rac1 was measured by monitoring the decrease in fluorescence at excitation/emission wavelengths of 485/535 nm on an ARVO X3 spectrofluorometer (PerkinElmer). The observed rate constants (kobs) of each reaction were determined through nonlinear least-squares fitting of the data with a single exponential decay model using KaleidaGraph software (Synergy Software).

### SPR

SPR experiments were performed using a Biacore T200 instrument (Cytiva). Rac1 (residues 1–188) was immobilized on a Series S Sensor Chip CM5 using the Amine Coupling Kit (Cytiva). Five different concentrations (0.37, 1.1, 3.3, 10, and 30 nM) of the full-length DOCK5^FL^/ELMO1^FL^ complex were continuously injected in the presence and absence of 10 μM RhoG^Q61L^. Next, the responses were measured in buffer containing 10 mM HEPES (pH 7.5), 150 mM NaCl, 1 mM MgCl_2_, 100 μM GTP, and 0.005% surfactant P-20. Data were processed through single-cycle kinetic analysis using the manufacturer’s software. The kinetic parameters were estimated from three independent experiments.

### Statistical analyses

Data were presented as mean ± SD considering three independent experiments. Two samples were statistically compared using an unpaired two-sided Student’s *t* test; statistical significance was considered at *p* < 0.05.

## Data availability

The cryo-EM density maps and corresponding atomic models have been deposited to the Electron Microscopy Data Bank (EMDB) and the PDB, respectively, under the following accession codes: DOCK5/ELMO1 complex (EMD-36271, 8JHK), RhoG/DOCK5/ELMO1/Rac1 complex (EMD-60136, 8ZJ2), RhoG/DOCK5/ELMO1 focused map (EMD-38466, 8XM7), DOCK5/ELMO1^CTD^/Rac1 core−class 1 (EMD-60146, 8ZJI), class 2 (EMD-60147, 8ZJJ), class 3 (EMD-60148, 8ZJK), class 4 (EMD-60149, 8ZJL), and class 5 (EMD-60150, 8ZJM). The mass spectrometry data have been deposited to the ProteomeXchange Consortium *via* the jPOST. The accession numbers are PXD048862 for ProteomeXchange and JPST002477 for jPOST.

## Supporting information

This article contains supporting information.

## Conflict of interest

The authors declare that they have no conflicts of interest with the contents of this article.
