# Supplementary material for: RhoG facilitates a conformational transition in the guanine nucleotide exchange factor complex DOCK5/ELMO1 to an open state
Source: J Biol Chem. 2024 Jun 8;300(7):107459. doi: 10.1016/j.jbc.2024.107459 (PMC11267001; doi:10.1016/j.jbc.2024.107459)
Supplement: Supporting Information [file mmc4.pdf]

Supporting Information for

RhoG facilitates a conformational transition in the guanine nucleotide exchange factor complex DOCK5/ELMO1 to an open state

Mutsuko Kukimoto-Niino*, Kazushige Katsura, Yoshiko Ishizuka-Katsura, Chiemi Mishima-Tsumagari, Mayumi Yonemochi, Mio Inoue, Reiko Nakagawa, Rahul Kaushik, Kam Y. J. Zhang, Mikako Shirouzu*

*Corresponding authors. Email: kukimoto@riken.jp, mikako.shirouzu@riken.jp

Supporting Information Contents:

Figures S1 to S7

Tables S1 to S3

Movies S1 to S3

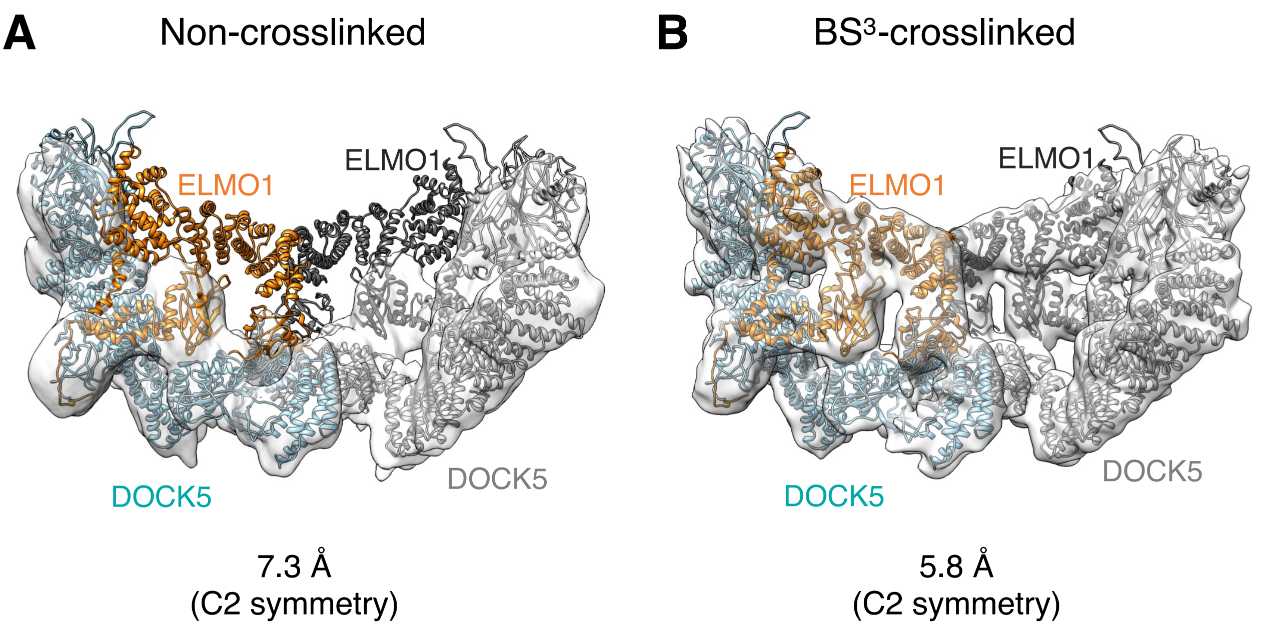

Fig. S1. Cryo-EM reconstructions from (A) non-crosslinked and (B) BS^3-crosslinked DOCK5/ELMO1 samples, showing that the $ELMO1^{NTD}$ is not ordered in the non-crosslinked sample. The final model obtained from the crosslinked sample was fitted to each map (one protomer in color, the other in grayscale).

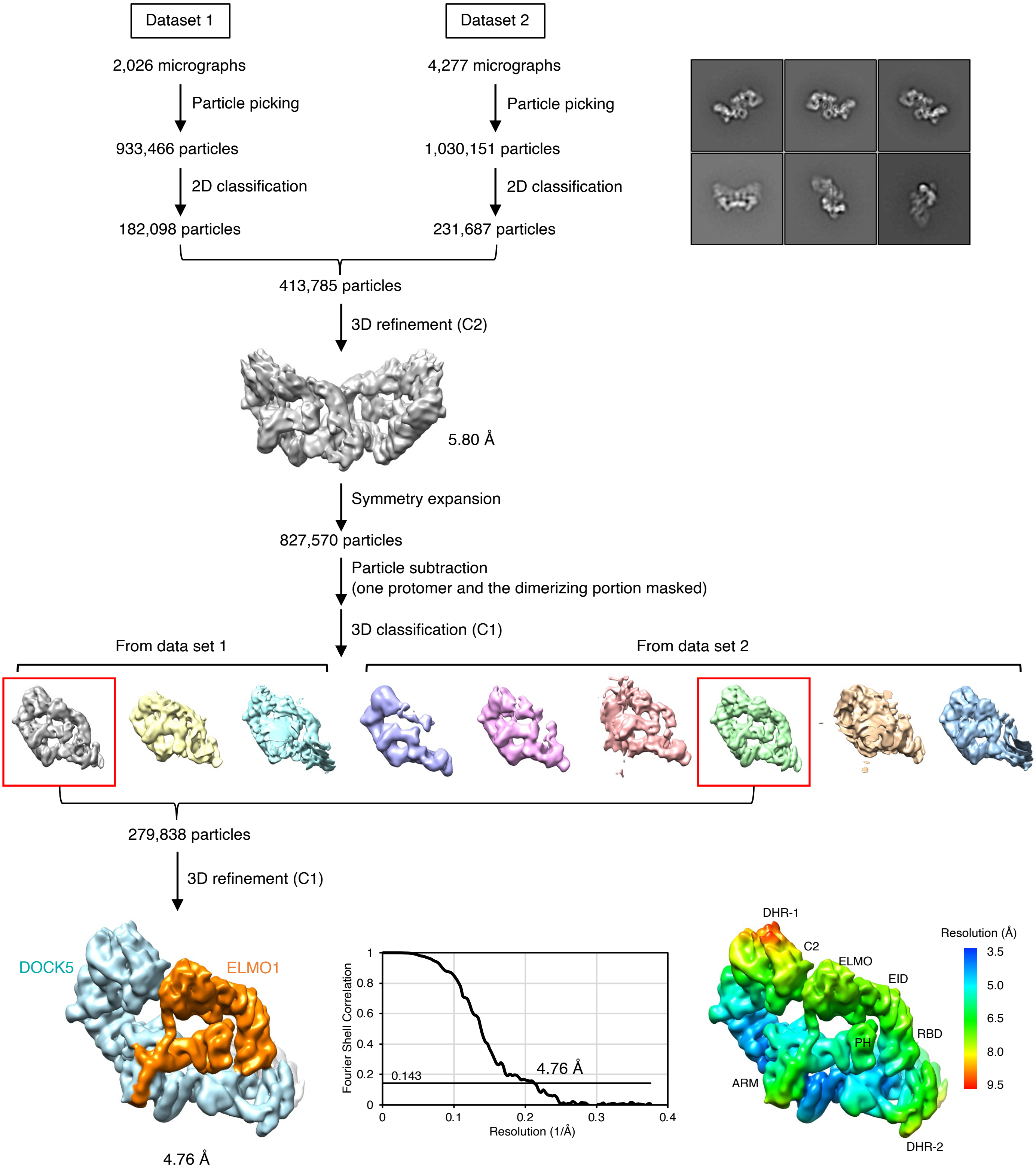

Fig. S2. Cryo-EM workflow of the DOCK5/ELMO1 complex.

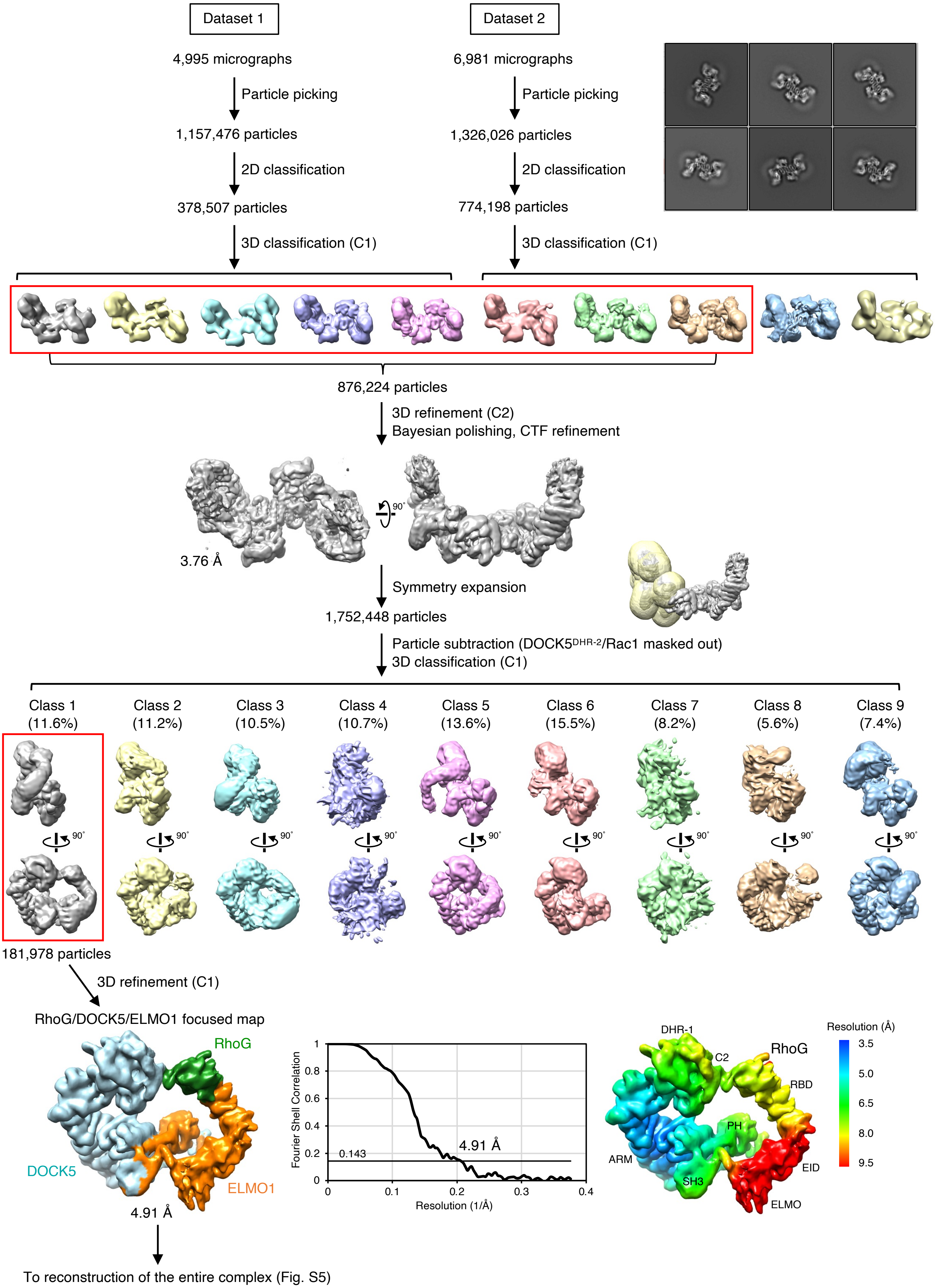

Fig. S3. Cryo-EM workflow of the RhoG/DOCK5/ELMO1/Rac1 complex focused on ELMO1^{NTD}.

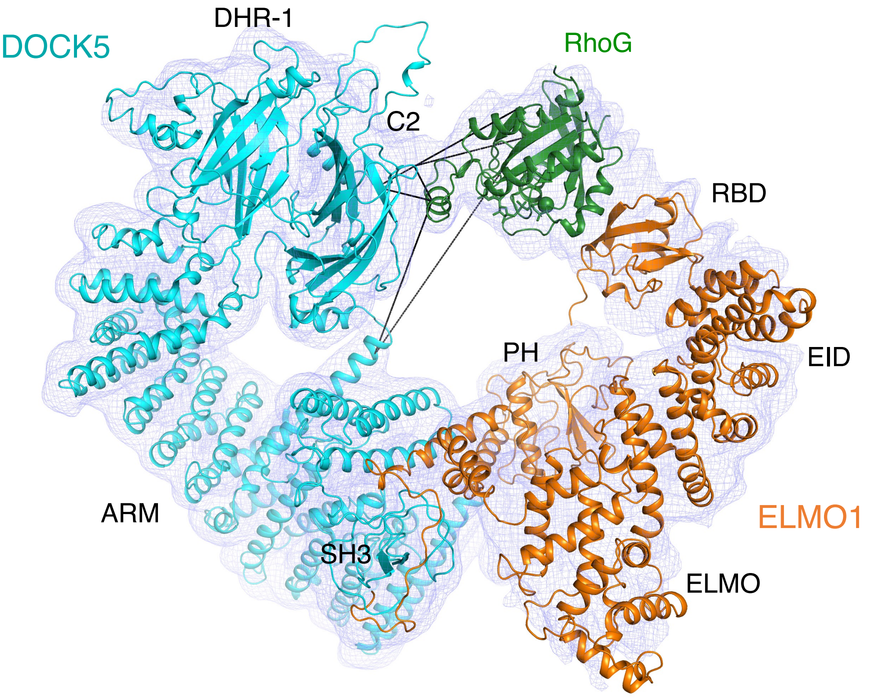

Fig. S4. The 4.9-Å cryo-EM map and atomic model of the RhoG/DOCK5/ELMO1 part show intermolecular BS^3 crosslinks detected across RhoG and DOCK5 (n = 3 independent experiments).

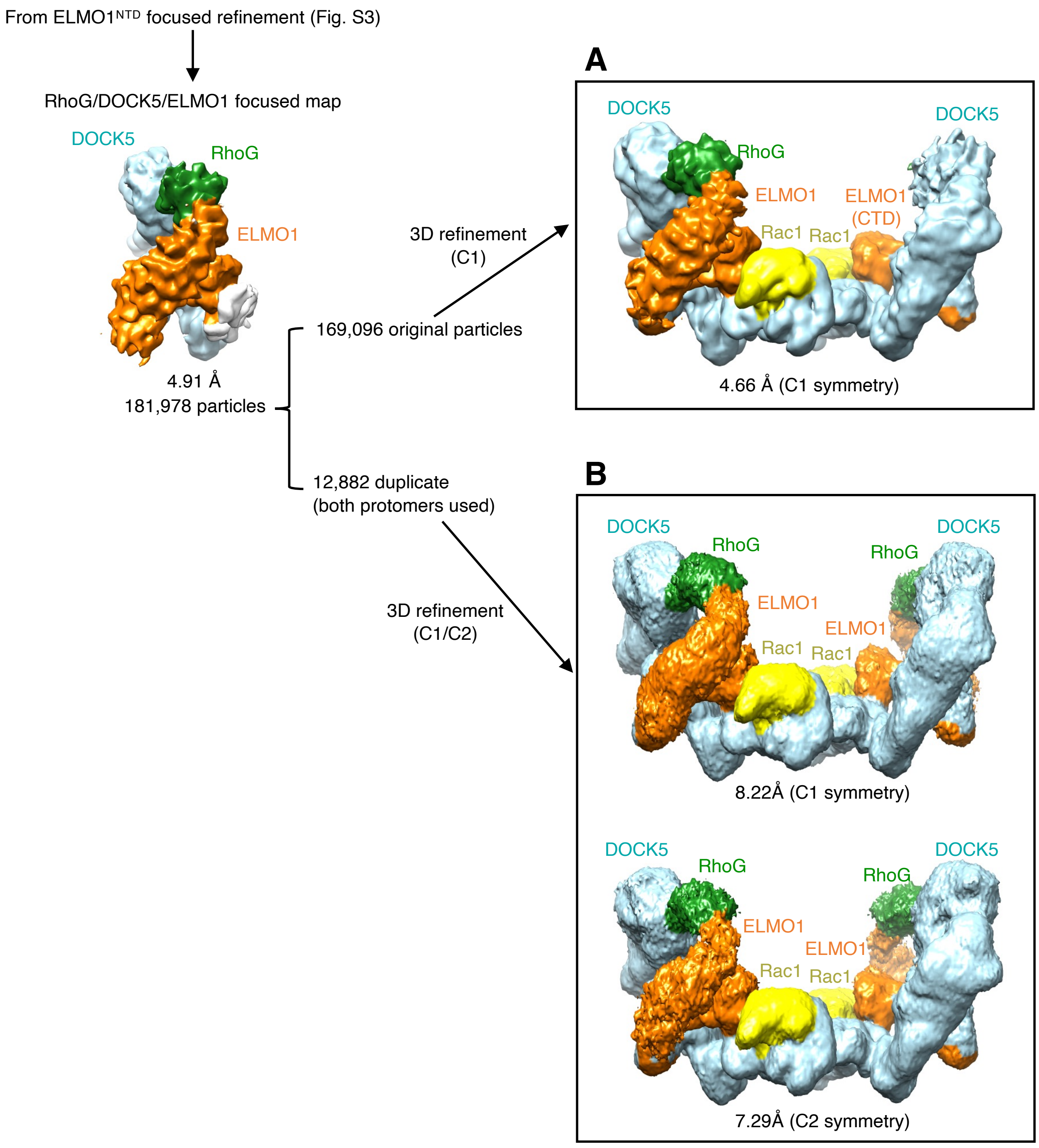

Fig. S5. 3D reconstructions of the entire RhoG/DOCK5/ELMO1/Rac1 complex. (A) Particles with a clear density for RhoG-ELMO1^{NTD} were reverted to the original particles to reconstruct the entire complex with one ordered RhoG-ELMO1^{NTD}. (B) Particles with both ordered RhoG-ELMO1^{NTD} were used to reconstruct the entire complex with C1 or C2 symmetry.

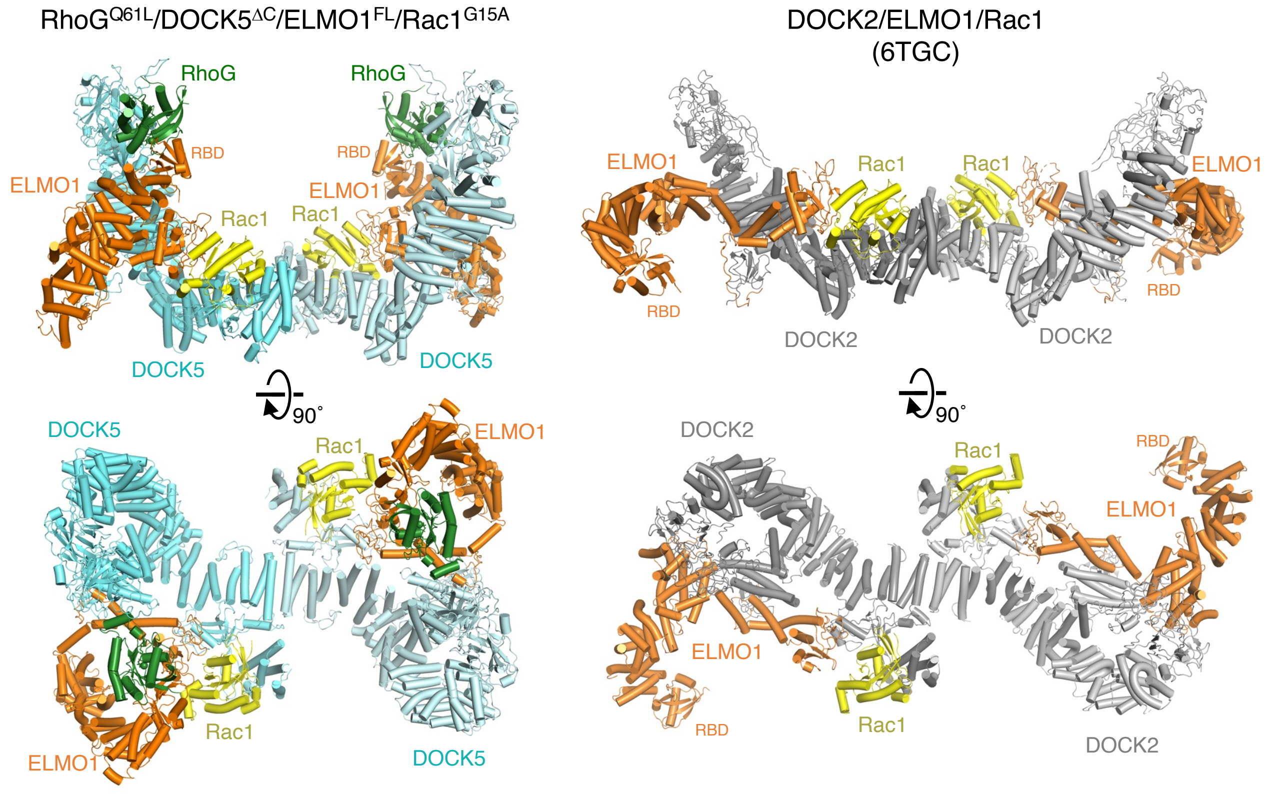

Fig. S6. Structural comparison of the RhoG/DOCK5/ELMO1/Rac1 complex (this study, left) and the DOCK2/ELMO1/Rac1 complex (PDB ID: 6TGC, right) in two orthogonal views.

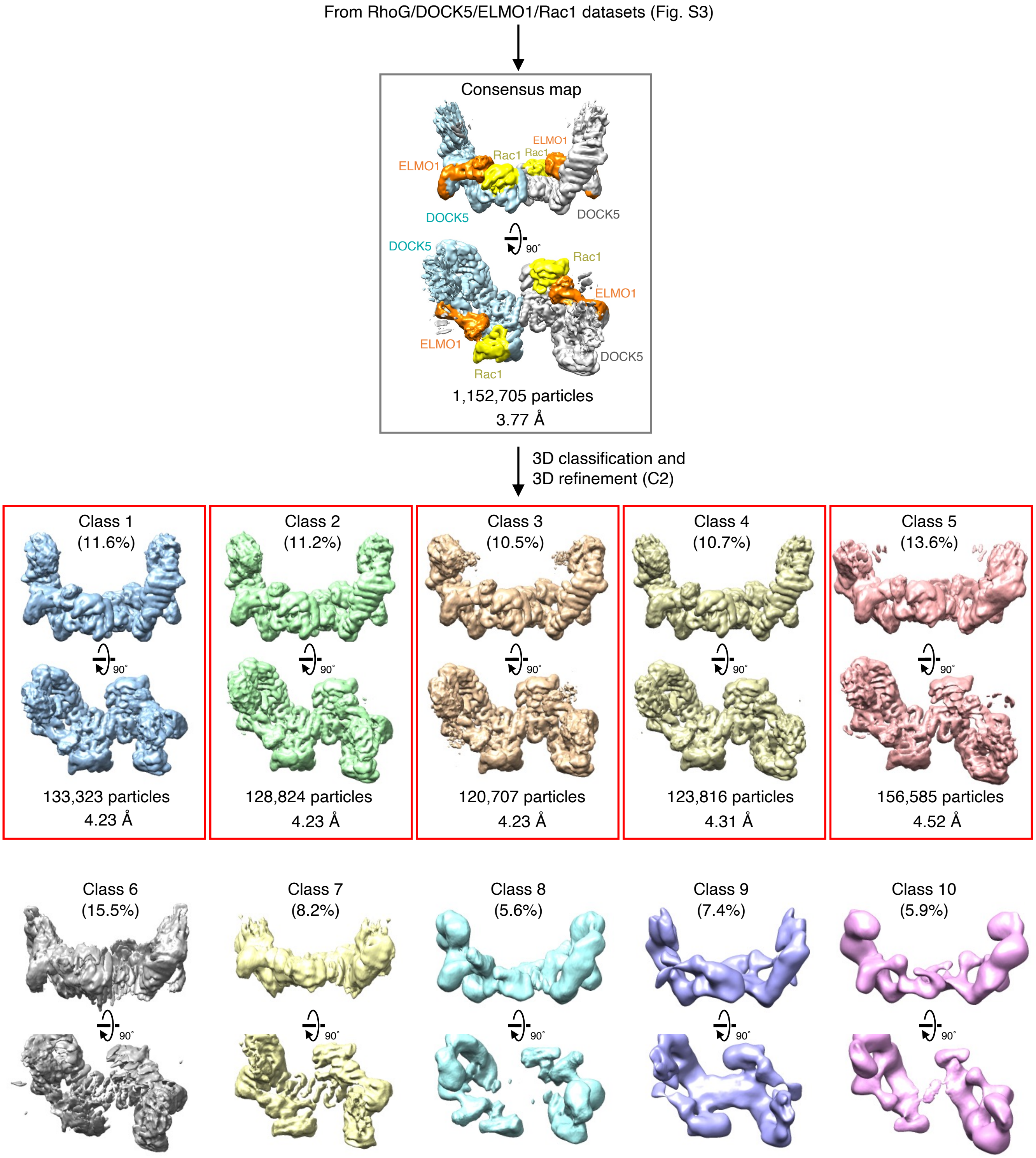

Fig. S7. 3D classification and refinement of the DOCK5/ELMO1^{CTD}/Rac1 core region from the consensus reconstruction of the RhoG/DOCK5/ELMO1/Rac1 complex.

Table S1. Cryo-EM data collection, refinement and validation statistics

	RhoG/DOCK5/ELMO1/Rac1 complex (EMD-60136) (PDB 8ZJ2)	RhoG/DOCK5/ELMO1 focused map (EMD-38466) (PDB 8XM7)	DOCK5/ELMO1 complex closed conformation (EMD-36271) (PDB 8JHK)
**Data collection and processing**			
Magnification	64,000	64,000	64,000
Voltage (kV)	300	300	300
Electron exposure ($e^{-}/Å^2$)	50	50	50
Defocus range (μm)	−0.8 to −2.0	−0.8 to −2.0	−0.8 to −2.0
Pixel size (Å)	1.33	1.33	1.33
Symmetry imposed	C1	C1	C1
Initial particle images (no.)	2,483,502	2,483,502	1,963,617
Final particle images (no.)	169,096	181,978	279,838
Map resolution (Å)	4.66	4.91	4.76
FSC threshold	0.143	0.143	0.143
**Refinement**			
Initial model used (PDB code)	6IE1, 7DPA, 7Y4A	6IE1, 7DPA, 7Y4A	6IE1, 7DPA
Model composition			
Non-hydrogen atoms	38,587	17,190	20,512
Protein residues	4,745	2,123	2,509
Ligands	GTP: 1, MG: 1	GTP: 1, MG: 1	–
B factors ($Å^2$)			
Protein	434.15	414.99	286.16
Ligand	999.99	766.19	–
R.m.s. deviations			
Bond lengths (Å)	0.006	0.007	0.006
Bond angles (°)	0.989	1.008	0.933
Validation			
MolProbity score	2.71	2.75	2.63
Clashscore	43.33	50.05	35.89
Poor rotamers (%)	0.00	0.00	0.00
Ramachandran plot			
Favored (%)	88.25	88.85	88.21
Allowed (%)	11.75	11.15	11.79
Disallowed (%)	0.00	0.00	0.00

Table S3. Cryo-EM data collection, refinement and validation statistics

DOCK5/ELMO1/Rac1 core	Class 1 (EMD-60146) (PDB 8ZJI)	Class 2 (EMD-60147) (PDB 8ZJJ)	Class 3 (EMD-60148) (PDB 8ZJK)	Class 4 (EMD-60149) (PDB 8ZJL)	Class 5 (EMD-60150) (PDB 8ZJM)
**Data collection and processing**					
Magnification	64,000	64,000	64,000	64,000	64,000
Voltage (kV)	300	300	300	300	300
Electron exposure (e^{-}/Å^2)	50	50	50	50	50
Defocus range (μm)	−0.8 to −2.0	−0.8 to −2.0	−0.8 to −2.0	−0.8 to −2.0	−0.8 to −2.0
Pixel size (Å)	1.33	1.33	1.33	1.33	1.33
Symmetry imposed	C2	C2	C2	C2	C2
Initial particle images (no.)	2,483,502	2,483,502	2,483,502	2,483,502	2,483,502
Final particle images (no.)	133,323	128,824	120,707	123,816	156,585
Map resolution (Å)	4.23	4.23	4.23	4.31	4.52
FSC threshold	0.143	0.143	0.143	0.143	0.143
**Refinement**					
Initial model used (PDB code)	7DPA	7DPA	7DPA	7DPA	7DPA
Model composition					
Non-hydrogen atoms	32,858	32,858	32,858	32,858	32,858
Protein residues	4,034	4,034	4,034	4,034	4,034
Ligands	–	–	–	–	–
B factors (Å^2)					
Protein	296.41	316.95	332.15	305.14	448.88
Ligand	–	–	–	–	–
R.m.s. deviations					
Bond lengths (Å)	0.005	0.005	0.007	0.005	0.006
Bond angles (°)	0.793	0.753	0.875	0.799	0.913
Validation					
MolProbity score	2.40	2.39	2.51	2.56	2.73
Clashscore	31.45	28.83	35.44	36.47	49.56
Poor rotamers (%)	0.00	0.00	0.00	0.00	0.00
Ramachandran plot					
Favored (%)	93.61	93.21	92.27	91.05	89.81
Allowed (%)	6.39	6.79	7.73	8.95	10.19
Disallowed (%)	0.00	0.00	0.00	0.00	0.00
